# Spinal osteoblastoma: a retrospective study of 35 patients’ imaging findings with an emphasis on MRI

**DOI:** 10.1186/s13244-020-00934-y

**Published:** 2020-11-23

**Authors:** Jianfang Liu, Songbo Han, Jie Li, Yuan Yuan, Wei Guo, Huishu Yuan

**Affiliations:** Department of Radiology, Peking University Third Hospital, 49 North Garden Road, Haidian District, Beijing, 100191 People’s Republic of China

**Keywords:** Osteoblastoma, Multimodal imaging, Spine, Edema

## Abstract

**Objective:**

To investigate the values of multimodal imaging approaches in the diagnosis of spinal osteoblastomas with an emphasis on MRI findings.

**Materials and methods:**

We retrospectively evaluated the imaging findings of 35 patients with spinal osteoblastomas. The imaging methods included radiography, whole-body bone scintigraphy (WBBS), CT and MRI.

**Results:**

Radiography detected 87.1% (27/31) of the lesions; WBBS demonstrated increased radionuclide activity in all the lesions. CT could precisely show and localize all niduses, and calcification was always detected. MRI usually could adequately delineate the niduses of osteoblastomas, especially on T2WI (88.2%; 30/34). 71.9% (23/32) of osteoblastomas were surrounded with moderate or extensive bone marrow edema (BME) with soft tissue edema (STE). STE always extended along the muscle bundle adjacent to the lesion; there was no subcutaneous fat involvement. BME was eccentrically distributed in the vertebral body and spread inward from the sides of the nidus. The extent of BME in the vertebral body tended to be inversely proportional to the distance from the nidus. In addition, rare magnifications of osteoblastoma including multifocal diseases (*n* = 2), vertebra plana (*n* = 1) or with aneurysmal bone cysts (*n* = 6) were also observed in our study.

**Conclusions:**

In patients showing moderate or extensive BME together with STE on MRI, both CT and MRI should be used to confirm nidus presence. The above-mentioned characteristics of edema on MRI of patients with spinal osteoblastoma are helpful in not only localizing the nidus, but also enhancing the diagnostic confidence.

## Introduction

Osteoblastomas are rare and locally aggressive intermediate tumors that account for approximately 1% of all primary bone tumors [1, 2]. The imaging features of osteoblastomas are largely anecdotal since the related literature is restricted to either case reports or small case series. The largest study on multimodal imaging approaches for diagnosing osteoblastomas was reported (Nemoto et al. in 1990 [3]). The study included 75 cases of osteoblastoma of the spine with 75 radiographs and 33 CT images; however, only two cases of diagnosis using MRI were available. MRI, which provides excellent soft tissue resolution, has played an increasingly important role in the diagnosis of diseases of the skeletal and muscular systems. Nevertheless, its value for the diagnosis of osteoblastomas remains controversial. Some authors recommend routine preoperative imaging of spinal osteoblastoma with CT and MRI in all cases [4]. Others claimed that MR imaging of osteoblastoma can be misleading with peritumoral inflammation mimicking malignant behavior [5, 6], or may overestimate the extent of the lesion due to extensive reactive changes and adjacent soft tissue masses [7]. To our knowledge, Shaikh et al. reported 11 cases of spinal osteoblastoma, which may be the largest number of such cases in which MRI application has been reported [7]. So, the conclusions of previous studied should be interpreted with caution since the sample size is relatively small in these series. It is very important to further explore the MRI features of spinal osteoblastoma in a larger sample study. To this end, in this study, we investigate the values of multimodal imaging approaches in the diagnosis of spinal osteoblastomas with an emphasis on MRI findings.

## Materials and methods

### Patient population

The images of 35 patients with osteoblastoma confirmed by pathology combined with radiology (the lesion larger than 20 mm in diameter on CT [1]) in our hospital between July 2006 and December 2019 were retrospectively analyzed. No patient had undergone any treatment before the imaging examination, which was conducted 1 week prior to biopsy or resection.

From July 2006 to December 2019, 1980 consecutive patients with tumors of the spine visited our institute. Among these cases, 874 were of original bone tumors and 59 of spinal osteoblastoma. Of these cases, 18 that had been previously treated at another institution and then referred to our institution for further evaluation and/or definitive treatment were excluded. Imaging data were unavailable in six cases. Finally, 35 patients with osteoblastoma were included in this study. The male-to-female ratio was approximately 2.89:1 (26 male and 9 female patients). The median age at presentation was 27 (8–58) years old.

### Imaging examination

Plain-film radiography and whole-body bone scintigraphy (WBBS) were performed in 31 and 13 cases, respectively**.** WBBS was performed with a delay of 3 h after the administration of the intravenous contrast medium, 99^m^Tc-methylene diphosphonate (99^m^Tc-MDP).

Plain CT scans, which were available for all cases (*n* = 35), had been performed with a 3-mm section thickness and 3-mm reconstruction interval. CT scans were obtained with one of the following commercially available devices: General Electric (GE) LightSpeed VCT, GE Discovery CT750 HD, Siemens Somatom, or Siemens Somatom Definition Flash.

MRI scans were obtained in 32 patients (34 niduses), with one of the following commercially available devices: GE Signa HDXT, GE Discovery MR 750, Siemens Sonata, or Siemens Magnetom Trio Tim 3.0 T. MRI sequences included T1-weighted images (T1WI, *n* = 32), T2-weighted images (T2WI, *n* = 32), and fat-suppressed T2-weighted images (FS T2WI, *n* = 32). For 17 patients (18 niduses), gadolinium contrast-enhanced FS T1WI sequences were available.

### Evaluation

The imaging data were evaluated retrospectively and independently by two experienced board-certified radiologists. Any difference of opinion was resolved by consensus between the two.

The following imaging features were analyzed. Plain-film radiography images were checked to determine whether the lesion could be observed or not and whether the lesion was an osteosclerotic, radiolucent, or mixed lesion. WBBS images were evaluated to determine whether a lesion showed intense uptake or not. CT images were analyzed to determine the location of the lesion, lesion morphology and size (the size refers to the longest diameter of a lesion three-dimensionally), and patterns of local extension. In addition, the images were analyzed for the presence or absence of a pathologic fracture, sclerosis of the peripheral margin of the lesion, mineralization of the tumor matrix, bony shell, whether the border of the nidus was well or poorly defined. MRI findings were analyzed to determine the predominant signal intensity characteristics of the nidus, and signal intensities were compared with those of the skeletal muscle. Presence and pattern of bone marrow edema (BME) and soft tissue edema (STE; grade, distribution, and extent) were evaluated on FS T2WI. Presence of calcification and a well-defined border was also analyzed.

Edema patterns were graded based on a previously described system [8]: Grade 1 (None): no perinidal edema. Grade 2 (Minor): thin rim of perinidal edema. Grade 3 (Moderate): edematous change circumferentially distributed around the nidus Grade 4 (Extensive): extensive edema more than in grade 3.

### Statistical analysis

For categorical variables, rates were compared using the Chi-square test. None of the variables conformed to a normal distribution; hence, we used median values (mix, max) to describe them. Statistical analyses were performed using the Wilcoxon (W) test for size comparison of the niduses measured on CT and MRI. Differences were considered significant when the P-value was less than 0.05. Statistical analysis was conducted with SPSS (version 19.0; SPSS Inc., Chicago, IL, USA).

## Results

Plain-film radiographs were available in 31 cases. Abnormal findings were noted for 27 patients; no abnormal findings were noted for the remaining four patients. Twenty-nine percent (9/31) of the niduses showed central lucency with a variable zone of peripheral sclerosis; 35.5% (11/31) were osteosclerotic and 22.6% (7/31) were radiolucent.

Scintigraphy in all the 13 cases demonstrated increased radionuclide activity in the affected area.

**Fig. 1:**
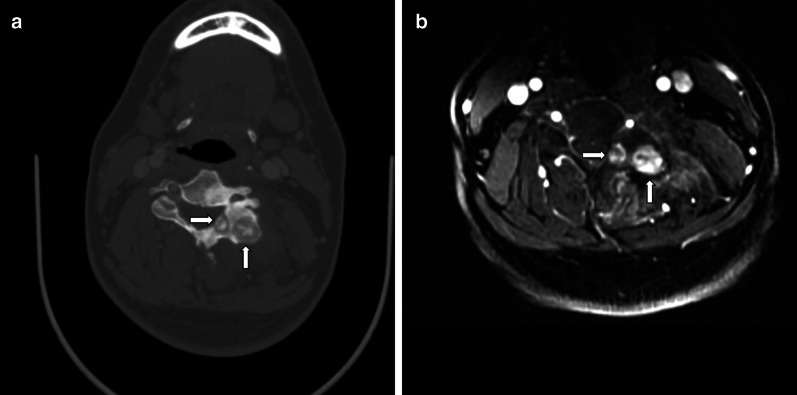
A 31-year-old man with multifocal lesions. **a** Shows representative images of with a lesion on the left accessory of C4. CT shows two obvious niduses in the accessory. Contrast enhancement on MRI can show the lesions well, with both the nidus and surrounding edema exhibiting intense enhancement (**b**)

**Fig. 2:**
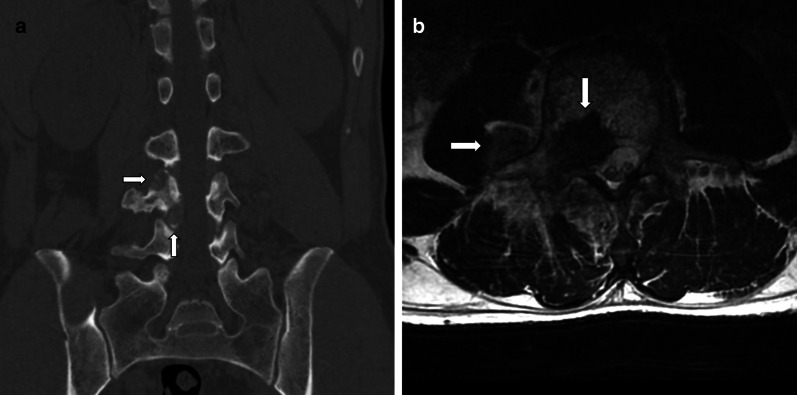
A 44-year-old man with multifocal lesions on the right accessory of L4 that involved to the vertebral body. CT shows two obvious niduses in the right accessory (**a**). An isointense signal nidus and a slight hyperintense signal nidus can be shown on T2WI (**b**)

**Fig. 3:**
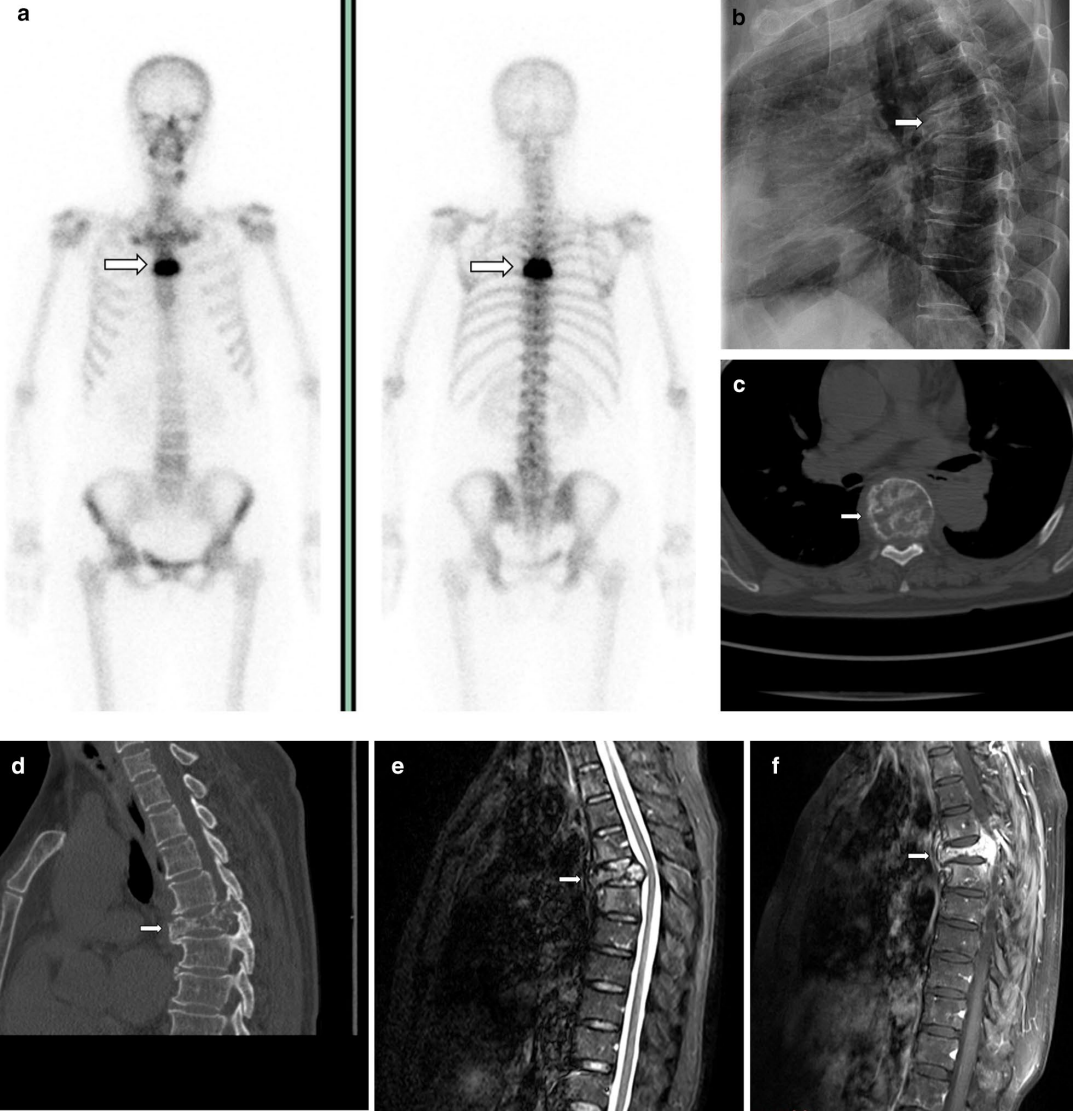
A 42-year-old man with osteoblastoma appearing as vertebra plana. Bone scan shows increased radionuclide tracer uptake at T7 (**a**). Radiograph shows a vertebra plana at T7 (**b**). CT depicts an expansile, mainly lytic soft tissue replacement of the bony trabeculae throughout the body (**c**, **d**). Pre- and paravertebral soft tissue extension, circumferential epidural extension enveloping and compressing the cord showed on T2WI (**e**) and contrast-enhanced T1WI (**f**)

**Fig. 4:**
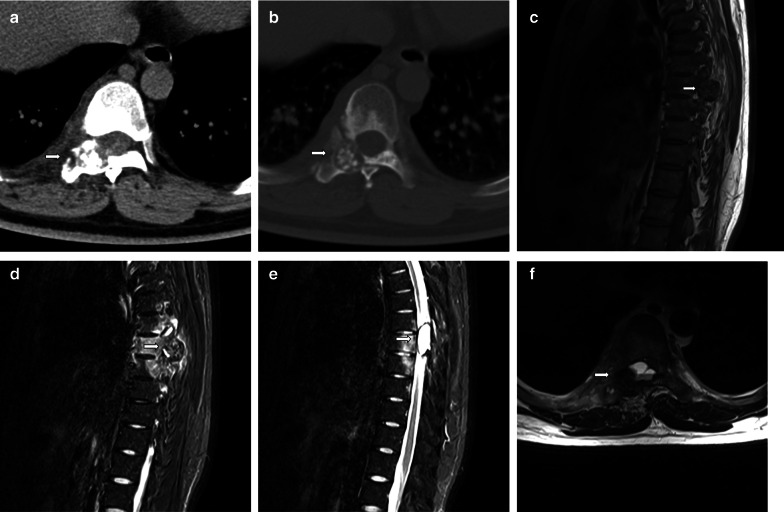
A 28-year-old man with aneurysmal bone cyst (ABC). A 28-year-old man with a lesion on the right accessory. The nidus can be visualized well on axial CT (**a**, **b**), especially in the bone window (**b**). The nidus exhibits isointense signal on T1WI (**c**) and hyperintense signal on FS T2WI (**d**). ABC appears conspicuous on MRI (**e**, **f**), with a typical finding of the fluid–fluid level (**f**)

Table 1 summarizes the CT findings. All niduses were larger than 20 mm in our study, except for one lesion in a multifocal osteoblastoma patient with the diameter of 9 mm. Generally, multifocal osteoblastoma is diagnosed with at least one lesion ≥ 20 mm [9]. Thus, multifocal osteoblastoma was diagnosed in two cases (Figs. 1, 2). As a result, 37 niduses were detected in 35 cases in our study. The typical findings on CT were a round or an oval region of bony destruction, with varying degrees of calcification, a thin bony shell and perinidal bone sclerosis. In most of the patients, 86.4% (32/37), the nidus was located in the accessory. In the remaining five patients (13.6%), the nidus was mainly located in the vertebral body. In two of the five cases, compression fractures were noted, including a serious one, indicated by the vertebral plana (Fig. 3); in another case, an aneurysmal bone cyst (ABC) was noted. In addition, there were six osteoblastomas accompanied by an ABC in total, which were detected remarkably on MRI (Fig. 4).

**Table 1:** CT findings

CT findings		Value
Location	CP/TP/LP	23/8/6
Center of nidus	Accessory/vertebral body	32/5
Size of nidus (mm)	Median (range)	30.1 (20–51)
Morphology	Round or oval/irregular	25/12
Destruction type	Osteogenic/lytic/mixed	14/14/9
Border	Well defined/poorly defined	16/21
Adjacent bone sclerosis	Yes/no	30/5
Bony shell	Yes/no	25/12
Calcification	Yes/no	37/0

CP/TP/LP: cervical vertebra/thoracic vertebra/lumbar vertebra

Osteogenic: Osteogenic > Lytic

Lytic: Osteogenic < Lytic

Mixed: Osteogenic = Lytic

Table 2 summarizes the MRI findings. T1WI, T2WI, FS T2WI, and contrast enhancement could recognize 85.3% (29/34), 88.2% (30/34), 85.3% (29/34), and 83.3% (15/18) of the niduses, respectively. One nidus was only recognized on T1WI, two niduses were only recognized on T2WI, and one nidus could only be recognized on contrast-enhancement images. When the findings for all MRI sequences were combined, all niduses were identified on MRI. The size measured on MRI showed no significant difference from the measurements on CT (*p* = 0.384).

**Table 2:** MRI findings

MRI findings		Value
Size of nidus (mm)	Median (range)	29.1(20–50)
Calcification	Yes/no	18/16
Border	Well defined/poorly defined	21/13
Signs of TIWI	Slightly hypointense/isointense	21/13
Signs of T2WI	Slightly hyperintense/hypointense	18/16
Signs of FS T2WI	Slightly hyperintense/hypointense	34/0
Enhanced degree of nidus	Slight/moderate/intense	4/6/8
Enhanced degree of edema	Slight/moderate/intense	0/0/17
BME	Yes/no	30/2
LSTE	Yes/no	29/3
Subcutaneous fat edema	Yes/no	0/32

BME: bone marrow edema; STE: soft tissue edema

**Fig. 5:**
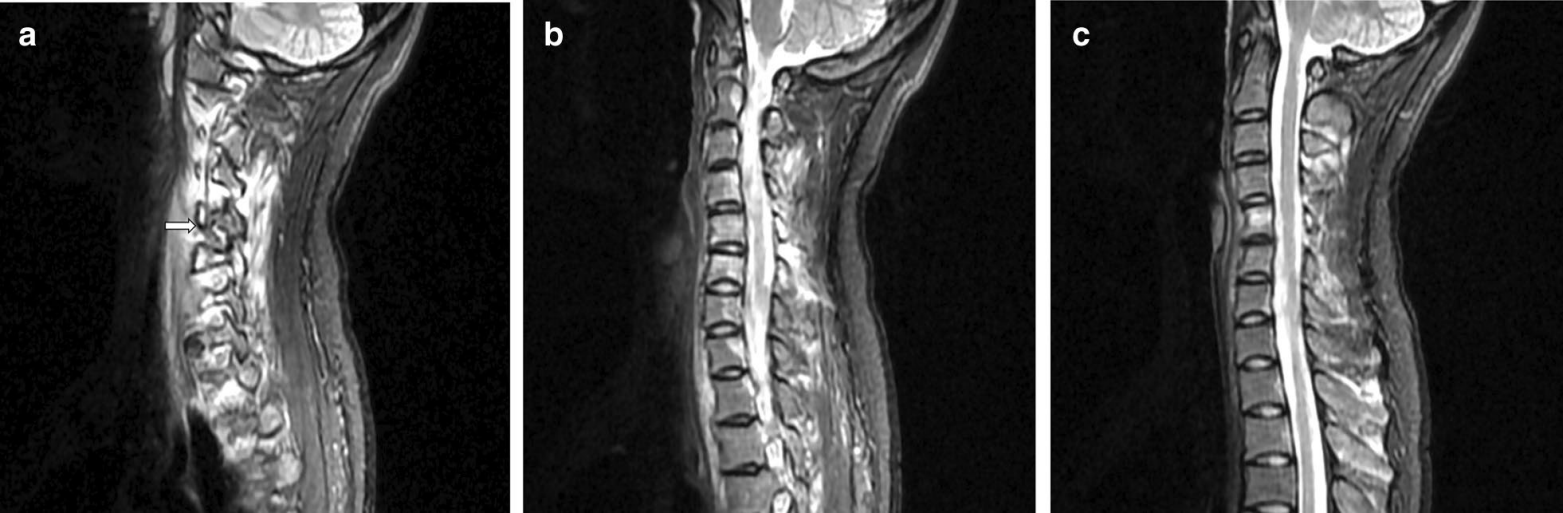
A 24-year-old woman with characteristic edema on FS T2WI. **a** Shows a lesion on the left accessory of C6. Both the soft tissue edema (STE) and bone marrow edema (BME) are evaluated as Grade 4, and the STE does not affect the subcutaneous fat. **b**, **c** Show the middle and right sides of the vertebra, respectively. BME in the vertebral body with eccentric distribution (**b**). The BME spreads inward from the side of the nidus (b) and sometimes affects the entire vertebral body (**b**, **c**). The vertebra containing the nidus showing the largest BME (**a**, **b**); the size of the BME in other vertebra is inversely proportional to the distance from the nidus

Table 3 shows the severity of edema. Reactive edema is a remarkable sign in osteoblastoma (Fig. 5), and STE and BME showed an incidence of 90.6% (29/32) and 93.7% (30/32), respectively, while 87.5% (28/32) of patients showed BME together with STE. In addition, 71.9% (23/32) of patients showed moderate or extensive BME together with STE. STE always spread along the muscle bundle adjacent the lesion, and none of the patients in our series showed involvement of the subcutaneous fat in edema. Although the lesions were usually located in the accessory, BME extended to the vertebral body in 83.3% (25/30) of the cases. A total of 84.4% (27/32) patients had BME in an average of 4 (range 1 − 11) vertebral bodies in our series. BME in the vertebral body showed some characteristic features (Fig. 5). BME in the vertebral body showed an eccentric distribution, with the BME spreading inward from the side of the nidus, and sometimes affecting the whole vertebral body. The vertebra containing the nidus showed the largest extent of BME, while the extent of the BME in other vertebrae was inversely proportional to the distance from the nidus. In addition, when the lesions were located in C1 (*n* = 1), C2 (*n* = 3) or spinous process (*n* = 3), the edema features were not typical. Among these three lesions in C2, one of them showed no edema, one case showed only STE (Grade 2), and one showed thin BME and STE (Grade 2). The lesions in C1 showed BME and STE in the distant vertebral body (C5-7) instead of the adjacent. Among these three lesions in spinous process, two of them showed edema involved the spinous process only and one case showed edema in the spinous process combined with distant vertebral body.

**Table 3:** Severity of edema

	Grade 1	Grade 2	Grade 3	Grade 4	Sum
BME	2 (6.3%)	3 (9.4%)	18(56.3%)	9 (28.1%)	32
STE	3 (9.4%)	3 (9.4%)	12 (37.5%)	14 (43.8%)	32

*BME* bone marrow edema, *STE* soft tissue edema

## Discussion

The choice of imaging technique to evaluate the presence of a solitary bone lesion depends on its accuracy, clinical acceptance, and influence on treatment options. Multimodality imaging reports of spinal osteoblastoma are relatively rare. We herein present the multimodality imaging features of spinal osteoblastoma in 35 patients, with an emphasis on the features in MRI.

Plain radiographs are the first choice for preliminary assessments in cases involving spinal surgery. Plain radiographs identified 87.1% (27/31) of the lesions in patients with spinal osteoblastoma in our series. However, plain radiography may fail to depict small lesions in complex anatomical areas such as the spine, where superimposed bony structures can obscure the lesion. The osteoblastoma typically appears as a central lucency with a variable zone of peripheral sclerosis [10], which was only shown in 29.0% of the cases in our series. We agree with the conclusion by Yin et al. that using radiography as the only diagnostic tool may result in a number of misdiagnoses [11]. Therefore, plain radiography is not a reliable diagnostic method of spinal osteoblastoma.

WBBS can detect all the lesions in our series. It is the most sensitive diagnostic tool described in the literature for osteoblastoma [12]. Nemoto et al. proposed that if the lesion is already discernible on a radiograph, WBBS is unnecessary [3]. Osteoblastomas are usually solitary lesions and are only rarely multicentric. In a review of the English literature, only three studies reported four cases of patients with multifocal osteoblastoma [13]. Our series included two patients with two synchronous lesions in a vertebra, which account for 5.7% (2/35) of all the cases. However, while multifocal lesions can appear as more than one nidus within a single bone, they may also involve more than one bone either synchronously or metachronously [9, 14]. In these few cases, the curative surgical procedure is more difficult. The patients’ pain may persist postoperatively if a tiny nidus is overlooked [9, 14, 15]. Therefore, WBBS may be an essential procedure for patients with osteoblastoma.

CT should be the investigation of choice for the characterization of suspected spinal osteoblastomas [7], since it can demonstrate and precisely localize the nidus. The most superior aspect of CT is its sensitivity for the detection of subtle calcification and the ability to reveal the types of bone destruction. Calcification is a typical finding of osteoblastoma, which were found in all of the lesions in our study. MRI showed low efficiency in detecting the calcification within the tumor in comparison with CT in our study, which is consistent with the previous reports [16, 17]. Thus, CT plays an irreplaceable role in the diagnosis of osteoblastoma.

Most osteoblastomas occur in the posterior elements and rarely in the vertebral body [18, 19]. In our study, five lesions were mainly located in the vertebral body, two of which presented with a compression fracture. One of the compression fracture cases appeared as a vertebra plana (Fig. 3). In 2017, Maharajan et al. [20] also reported an osteoblastoma presenting as vertebra plana and proposed to add spinal osteoblastoma as one more etiology for vertebra plana. Another case appeared as ABC; there were six cases of osteoblastoma with ABC in our study. An association between osteoblastoma and ABC was observed in 16.2% (6/37) of the cases of our series, which is consistent with the value reported by Della Rocca and Huvos in 1996 (14.5%) [21]. ABC is better characterized on MRI than on CT.

The role of MRI in the diagnosis of osteoblastoma has always been controversial. With the advantage of excellent soft tissue resolution, MRI has played an important role in identifying spinal cord and nerve root compression, thus influencing the option of treatment method and predicting prognosis for patients with spinal osteoblastoma. Some authors recommend routine preoperative imaging of spinal osteoblastoma with CT and MRI in all cases [22]. However, the nidus of an osteoblastoma has a very heterogeneous, variable appearance on MRI, making characterization difficult [7]. Reactive edema, including BME and STE, is the most remarkable finding on MRI in patients with osteoblastoma. 93.7% (30/32) of osteoblastoma patients presented with BME and 90.6% (29/32) of osteoblastoma patients were diagnosed with STE. Surrounding edema occurred in 86.4%–90.9% of patients with osteoblastoma as reported by previous studies, which was comparable to our finding in this study. Peritumoral inflammation has been commonly associated with osteoblastoma and is thought to be secondary to prostaglandin production by the tumor [23]. Some authors claim that MR imaging of osteoblastoma can be misleading, with peritumoral inflammation mimicking malignant behavior [5, 6]. Gao and Kroon H.M [24, 25] noted that edema was not a specific indicator of malignant bone tumors, and it was also associated with benign bone tumors and tumor-like diseases. Although many kinds of primary bone tumors may be surrounded with edema in the background, only a few of them showed moderate or extensive BME together with STE. According to Kroon H. M’s report of 69 cases with 13 kinds of bone tumors [25], only 15.4% (6/39) of the malignant bone tumors and 16.7% (4/24) of the benign bone tumors are surrounded with moderate or extensive BME together with STE. In our study, 71.9% (23/32) of osteoblastomas were surrounded with moderate or extensive BME together with STE. Therefore, we suggest that the presentation of moderate or extensive BME together with STE on MRI of the spine might be a reliable hint for the diagnosis of spinal osteoblastoma.

In addition, we also found that MRI shows edema of osteoblastoma with the following features, which was not reported in previous studies: STE always extended along the muscle bundle adjacent the lesion without subcutaneous fat involvement; BME in the vertebral body showed some typical characteristics, such as an eccentric distribution and a tendency to spread inward from the side of the nidus, sometimes affecting the whole vertebral body; the vertebra containing the nidus showed the largest BME, with the size of the BME in other vertebra being inversely proportional to the distance from nidus. This feature may indicate the approximate location of the nidus. In addition, in our presentation, the lesions located in the C1, C2 and spinous process tended to not show these typical edema features.

Some authors claimed that MRI may overestimate the extent of the lesion due to extensive reactive changes and adjacent soft tissue masses [7]. As we have mentioned above, the presentation of moderate or extensive BME together with STE on MRI of the spine should raise our awareness of considering the diagnosis of spinal osteoblastoma. And additionally, the distribution features of the edema might provide information on finding the nidus. In our study, the niduses of osteoblastomas could be adequately delineated on MRI, especially on T2WI. The combination of all the plain scan sequences on MRI could differ niduses from edema in all except one case with multifocal niduses, which could only be distinguished in the contrast-enhancement images. The average size measured on MRI showed no significant difference from that on CT in our study. In other words, MRI could also estimate the extent of the lesion precisely.

Although the literature has reported marked enhancement in edema of osteoblastoma and homogeneous or heterogeneous enhancement in niduses [26], there are few detailed descriptions of imaging findings of enhanced MRI of spinal osteoblastoma. In our study, all the edema shows obvious homogeneous enhancement, 55.6% (10/18) of which exhibits low or moderate enhancement with a clear boundary. Therefore, its niduses are easy to be distinguished from edema in these cases. The remaining eight niduses showed obvious enhancement, five out of which could be also distinguished from edema based on the non-enhancement hypointense ring surrounding each nidus. In conclusion, 83.3% (15/18) of the niduses could be recognized on contrast-enhancement images. In our series, one nidus could only be recognized on contrast enhancement images. Contrast-enhanced MRI scan may be useful to distinguish niduses from the edema for patients suspicious of osteoblastoma.

Our study had several limitations. This was a retrospective review, which has inherent shortcomings but was unavoidable since the rare incidence of this tumor precludes prospective patient accrual. Another limitation is that imaging protocols were not strictly controlled, since our cases spanned 13 years and were evaluated with different kinds of equipment.

In conclusion, in patients showing moderate or extensive BME together with STE on MRI, both CT and MRI should be used to confirm nidus presence. The above-mentioned characteristics of edema on MRI of patients with spinal osteoblastoma are helpful in not only localizing the nidus, but also enhancing the diagnostic confidence.

## Data Availability

Information on where data supporting the results reported in the article can be found at Peking University Third Hospital.

## Abbreviations

ABC: Aneurysmal bone cyst
BME: Bone marrow edema
FS T2WI: Fat-suppressed T2-weighted images
STE: Soft tissue edema
T1WI: T1-weighted images
T2WI: T2-weighted images
